# Novel splicing‐site mutation in *DCAF17* gene causing Woodhouse‐Sakati syndrome in a large consanguineous family

**DOI:** 10.1002/jcla.24127

**Published:** 2021-12-08

**Authors:** Fozia Fozia, Khadim Shah, Rubina Nazli, Sher Alam Khan, Ijaz Ahmad, Noor Mohammad, Saadullah Khan, Amal Alotaibi

**Affiliations:** ^1^ Department of Biochemistry Institute of Basic Medical Sciences (IBMS) Khyber Medical University (KMU) Peshawar Khyber Pakhtunkhwa Pakistan; ^2^ Department of Biotechnology COSMATS University Islamabad Abbottabad Campus Abbottabad Khyber Pakhtunkhwa Pakistan; ^3^ Department of Biotechnology and Genetic Engineering Kohat University of Science & Technology (KUST) Kohat Khyber Pakhtunkhwa Pakistan; ^4^ Department of Chemistry Kohat University of Science & Technology (KUST) Kohat Khyber Pakhtunkhwa Pakistan; ^5^ Basic Science Department College of Medicine Princess Nourah Bint Abdulrahman University Riyadh Saudi Arabia

**Keywords:** *DCAF17*, splicing‐site variant, Woodhouse‐Sakati syndrome, WSS

## Abstract

**Background:**

Woodhouse‐Sakati syndrome is a rare autosomal recessive disease with endocrine and neuroectodermal aberrations with heterogeneous phenotypes and disease course. The most common phenotypes of the disease are progressive sensorineural hearing loss and alopecia, mild‐to‐moderate mental retardation and hypogonadism. The disease results from mutations in the *DCAF17* gene.

**Method:**

Here, we reported a large consanguineous pedigree with multiple affected individuals with Woodhouse‐Sakati syndrome phenotypes. Laboratory tests confirmed the endocrine perturbance in affected individuals. To find out the underlying genetic change, whole‐exome sequencing was carried out.

**Result:**

Analysis of the exome data identified a splicing‐site deletion NM_025000.3:c.1423‐1_1425delGACA in *DCAF17* gene. Sanger sequencing confirmed the co‐segregation of the variant with the disease phenotypes in the family.

**Conclusion:**

The variant is predicted to cause aberrant splicing, i.e., exon skipping, resulting in the translation of a truncated functionless protein which results in appearance of typical phenotypic features and clinical laboratory findings of Woodhouse‐Sakati syndrome in affected members of the family.

## Graphical abstract

Woodhouse‐Sakati syndrome, a rare autosomal recessive disease with endocrine and neuroectodermal abnormalities including progressive sensorineural hearing loss, alopecia, mental retardation and hypogonadism resulting from mutations in the *DCAF17* gene. Here we reported a large consanguineous family with multiple affected individuals showing Woodhouse‐Sakati syndrome phenotypes and having a novel a splice site deletion mutation in *DCAF17* gene

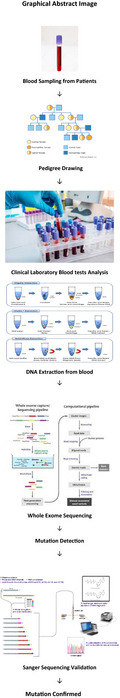

## 1 INTRODUCTION

Woodhouse‐Sakati syndrome (WSS; MIM 241080) is a rare disorder of neuroendocrine origin and is inherited in an autosomal recessive manner. It was reported for the first time by Woodhouse and Sakati (1983). The progressive disease features varied from person to person, even within the same family. Most of the affected individuals show neurological problems (sensorineural hearing loss, seizures, sensory polyneuropathy, dysarthria, dystonia, and magnetic resonance imaging (MRI) abnormalities), endocrine complications (hypogonadism, hypothyroidism, and diabetes mellitus), abnormal ectodermal structures (hair loss and anodontia), and electrocardiographic and craniofacial anomalies.^1^, ^2^, ^3^, ^4^, ^5^ Due to a defective endocrine system, affected individuals display developmental defects in secondary sexual characteristics. The hair loss in affected individuals begins in temporal and occipital regions in childhood that gradually worsens, often resulting in alopecia totalis during their adulthood. The Affected men have sparse or absent facial hairs, eyebrows, and eyelashes.^6^

The gene *DCAF17* (MIM 606119), also known as *C2ORF37*, which is considered the cause of WSS, encodes DDB1‐ and CUL4‐associated factor 17. The *DCAF17* gene, which is found on chromosome 2q31.1, produces two nucleolar proteins along with two isoforms, alpha (453 amino acid residues; NP 001158293.1) and a beta (520 amino acid residues; NP 079276.2).^6^, ^7^ In humans, it has ubiquitous expression, while in mice its prominent expression has been reported in the brain, liver, skin, and in seminiferous tubules, which is thought to be responsible for the neurosensory, endocrine, and cutaneous phenotypic expressions.^1^ Previous research on this nucleolar protein revealed that it served as a substrate co‐receptor for the ubiquitin ligase complex Cul4‐DDB1,^4^, ^8^ highlighting the importance of nucleolus in maintaining life functions, including cellular activities, such as growth, proliferation, stress response, along with cell cycle and apoptosis.^9^ The sequence of amino acids of the DCAF17 protein remains conserved across different species, including rat, mouse, cow, fowl, chimpanzees, and human beings. The first pathogenic sequence, variant in the *DCAF17* gene was identified by Alazami et al.^1^ in an Arabic family with WSS phenotypes. Subsequently, mutations in the same gene have been stated in different ethnic groups across the globe, including Arabic, Pakistani, Indian, Turkish, French, and Italian populations.^1^, ^2^, ^3^, ^4^, ^5^ Previously, three pathogenic sequence variants in Pakistani population have been identified for WSS phenotypes.^3^, ^4^, ^5^ In the present study, we identified a novel splicing‐site deletion variant in a large consanguineous Pashtun Pakistani inbreed, segregating with disease phenotypes.

## 2 MATERIALS AND METHODS

### 2.1 Study subjects

**FIGURE 1:**
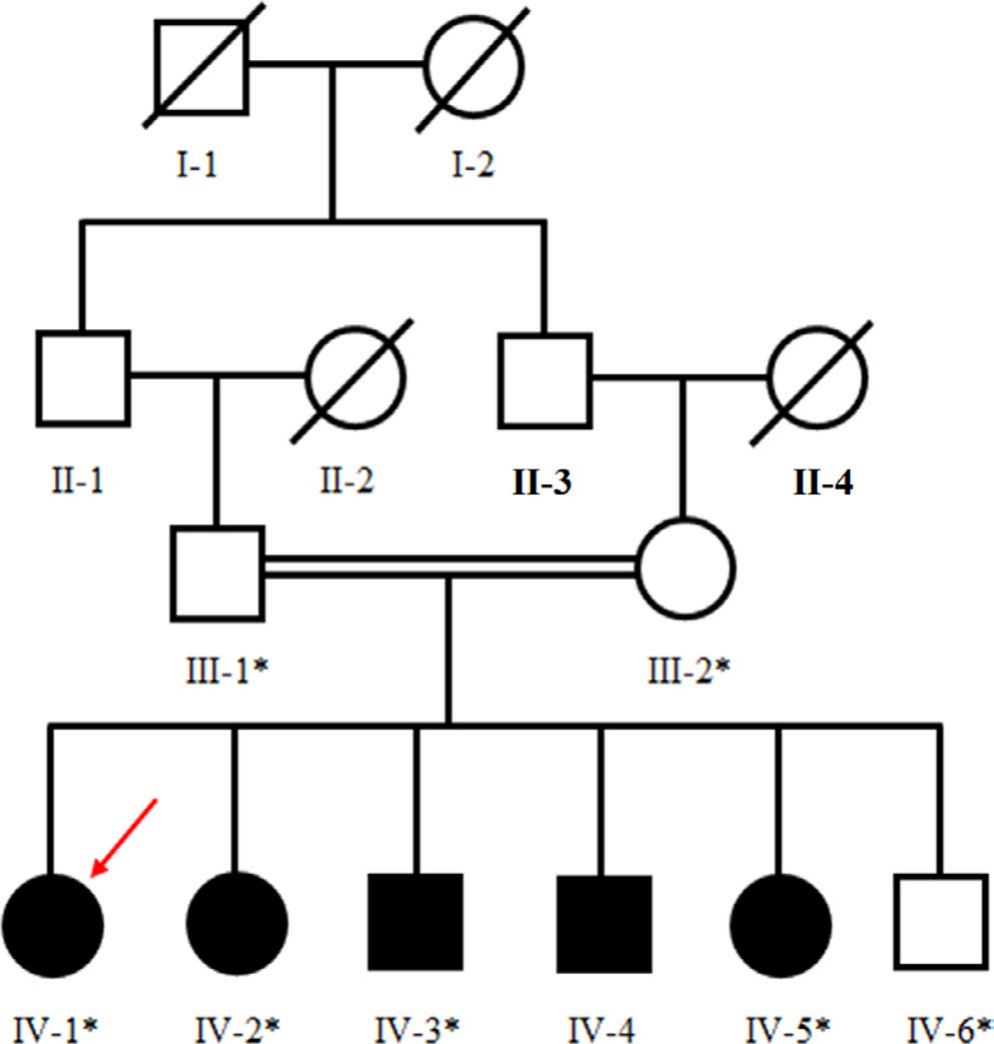
Pedigree of the family segregating Woodhouse‐Sakati syndrome phenotypes in an autosomal recessive manner. Individual who underwent whole‐exome sequencing is indicated by arrow

The studied four‐generation consanguineous pedigree has five affected individuals, including two males (IV‐3, IV‐4) and three females (IV‐1, IV‐2, and IV‐5), segregating autosomal recessive WSS phenotypes (Figure 1). Approval for this study was obtained from Institutional Review Board, of Khyber Medical University (KMU), Peshawar, and Kohat University of Science and Technology (KUST), Kohat, Pakistan. Informed written consent was obtained from all the participants. Detailed physical and clinical examination of affected members was carried out at a tertiary care hospital.

### 2.2 Samples collection and DNA extraction

For DNA extraction, peripheral blood samples were collected from all the participants in EDTA containing vacutainer tubes. Extraction of DNA from the whole blood was carried out by following a standard phenol‐chloroform protocol, as described somewhere else (Ullah et al., 2017).

### 2.3 Whole‐exome sequencing

DNA sample of an affected individual (IV‐1) was submitted to CENTOGENE for whole‐exome sequencing (WES). Briefly, exome enrichment was performed using the Twist Human Core Exome plus Kit. Sequencing with a minimum depth of 20× was performed on an Illumina platform for >98% of the targeted bases. The in‐house bioinformatics pipeline of CENTOGENE was used for alignment to GRCh37/hg19 genome assembly, variant calling, annotation, and variant filtering. Homozygous variants with a frequency of ≤1% in gnomAD database (https://gnomad.broadinstitute.org/) and exome sequencing project, (ESP; Exome Variant Server (washington.edu)), and disease causing variants reported HGMD (http://www.hgmd.cf.ac.uk/ac/all.php), in ClinVar (https://www.ncbi.nlm.nih.gov/clinvar/) or in CentoMD (https://www.centogene.com/pharma/mutation‐database‐centomd.html) were considered, focusing on coding exons and exon‐intron splicing nucleotides, as described in previous studies.^10^, ^11^, ^12^, ^13^, ^14^ For segregation analysis of the pathogenic variant, Sanger sequencing was performed as described earlier.^15^

## 3 RESULTS

### 3.1 Clinical findings

**FIGURE 2:**
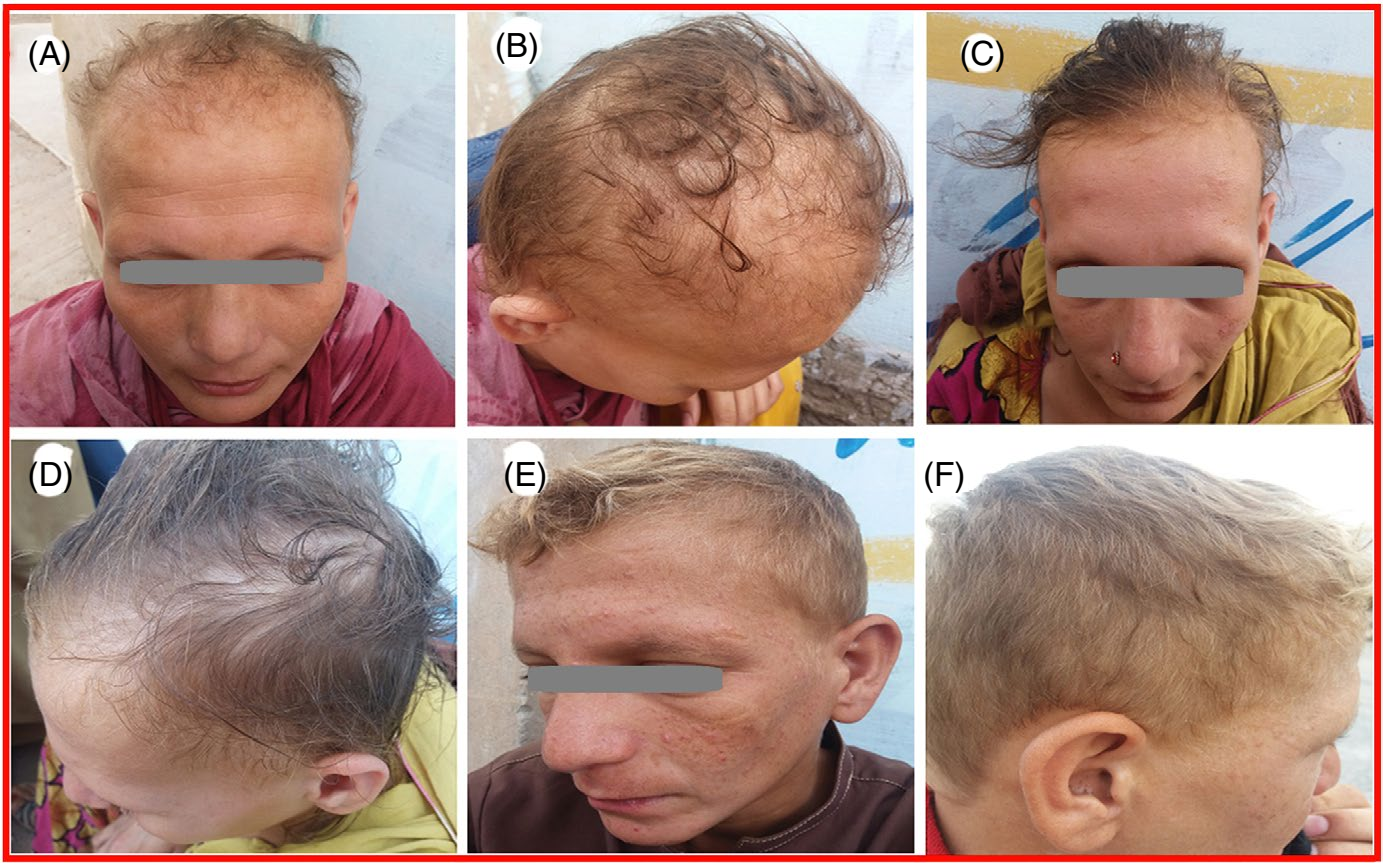
Clinical pictures of the affected individuals, Member IV‐1 (A, B), Member IV‐2 (C, D), and Member IV‐3 (E, F) demonstration typical features of WHS Syndrome, including Sparse, thin, short, Rough, dry, lighter in color scalp hairs, Facial dysmorphism, Triangular‐shaped elongated face, flat occiput, Prominent supraorbital ridge, Hypertelorism, Prominent nasal root, Failure of secondary sexual characters development, and precocious aging

A large consanguineous pedigree, including five affected siblings (IV‐1, IV‐2, IV‐3, IV‐4, and IV‐5), showing WSS phenotypes was studied. Specialized clinicians examination revealed presence of alopecia, intellectual disability, hypogonadism, mild sensory neural deafness, delayed speech, language development, and extrapyramidal features in affected members of the family (Table 1). Hair loss disorder in the affected patients was defined by the presence of sparse, thin, short, dry, and lighter in color scalp hairs that break easily, scanty eyebrows, and absence of axillary and pubic region hair. Nails and teeth are normal. They also display sensory neural deafness and intellectual disability of mild severity. Facial dysmorphism in the form of an elongated face, flat occiput, prominent supraorbital ridge, hypertelorism, and prominent nasal bridge were observed in affected members of the family (Figure 2). The affected female individuals (IV‐1, IV‐2) confirmed the occurrence of hypogonadism during puberty. During adolescence, both the sisters experienced failure of development of secondary sexual characters along with an infantile uterus, primary amenorrhea, and breast hypoplasia. They also exhibit precocious skin aging, early‐onset diabetes mellitus. Their ECG revealed flattened T waves. Biochemical investigation shows an increase in serum follicle‐stimulating hormone (FSH) and luteinizing hormone (LH) levels, and decreased prolactin, estradiol, and cholesterol levels. The glycated hemoglobin (HbA1C) levels are within the range of uncontrolled glycemia. The two affected male individuals (IV‐3 and IV‐4) demonstrate underdeveloped secondary sexual characters and external genitalia. Blepharospasm, decrease levels of IGF‐1, and increase serum cholesterol are illustrated (Table 1) in affected individual (IV‐3), while the other male patient (IV‐4) exhibits extrapyramidal manifestations as mentioned in previous studies.^2^, ^3^, ^4^, ^5^ He displayed myogenic contractures with stiffed muscles, blepharospasm, camptodactyly, spastic quadriplegia, choreoathetosis dysarthria, dystonia and dysphagia, microcephaly with a history of seizures since childhood along with incontinence. Radiological investigations, such as MRI and computed tomography (CT), of the affected individuals were not available for analysis. Previous family history of hypertension and diabetes is reported by the parents. The anticipated heterozygous carriers present in the family were asymptomatic and showed normal phenotypic and genotypic appearance.

**TABLE 1:** Clinical and laboratory features of affected individuals in studied family

Clinical features	Affected members				
ID in Pedigree	IV‐1	IV‐2	IV‐3	IV‐5	IV‐6
Sex	Female	Female	Male	Male	Female
Age (years)	18	16	14	11	9
Height (cm)	188	185	192	152	92
Weight (kg)	60	59	58	55	48
Ectodermal appendages					
Hair abnormalities					
Sparse, fine, short, and fragile scalp hair	+	+	+	+	+
Scanty eyebrows	+	+	+	+	+
Sparse eyelashes	−	−	−	−	−
Body hairs	Normal	Normal	Normal	Normal	Normal
Axillary hairs	−	−	−	NA	NA
Pubic hairs	−	−	−	NA	NA
Nails disorders	−	−	−	−	−
Anodontia	−	−	−	−	−
Hypoplasia of dental enamel	−	−	−	−	−
Precocious skin aging	+	+	−	−	−
Palmer and planter hyperkeratosis	−	−	−	−	−
Ocular findings					
Keratoconus	−	−	−	−	−
Neurological features					
Sensorineural deafness	Moderate	Mild	Mild	Moderate	Mild
Intellectual disability	Mild	Mild	Mild	Moderate	Mild
Extrapyramidal features					
Dystonia	−	−	−	+	−
Blepharospasm	−	−	+	+	−
Choreoathetosis	−	−	−	+	
Dysarthria	−	−	−	+	−
Dysphagia	−	−	−	+	−
Spastic quadriplegia	−	−	−	+	−
Myogenic contractures	−	−	−	+	−
Seizure	History in childhood	History in childhood	−	+	−
Delayed speech and language development	+	+	+	+	+
Microcephaly	−	−	−	+	−
Endocrine features					
Hypogonadism	+	+	+	+	+
Failure of secondary sexual characters development	+	+	+	+	+
Breast hypoplasia	+	+	−	−	−
Widen Intermammillary distance	+	+	+	+	+
Infantile Uterus with absent menstruation	+	+	−	−	−
Diabetes mellitus	+	+	−	−	−
Thyroid	Normal	Normal	Normal	Normal	Normal
Other features					
Facial dysmorphism—Triangular‐shaped elongated face	+	+	+	+	−
Flat occiput	+	+	+	+	+
Prominent supraorbital ridge	+	+	+	+	−
Hypertelorism	−	−	−	−	−
Prominent nasal root	+	+	+	+	−
Incontinence	−	−	−	+	−
Camptodactyly	−	−	−	+	−
Acanthosis nigricans	−	−	−	−	−
ECG changes flattened T wave	+	+	−	−	−
Laboratory tests					
IGF‐1 (ng/ml)	28.09	33.67	51.04		
Thyroxin (T4) µg/ml	8.87	11.62	9.64	NA	NA
TSH (µU/ml)	2.786	1.025	3.778	NA	NA
LH (mIU/ml)	18.55	24.32	6.95	NA	NA
FSH (mIU/ml)	77.64	59.03	4.26	NA	NA
PRL (ng/dl)	35.67	46.12	92.0	NA	NA
Testosterone (ng/ml)	NA	NA	10.3	NA	NA
Estradiol (pg/ml)	49.66	54.92	NA	NA	NA
hbA1C (%)	7.8%	7.6%	5.8%	NA	NA
Serum cholesterol (mg/dl)	294	288	263	NA	NA

Note

+, presence of feature; −, absence of sign; NA, not available.

Reference range of laboratory tests; IGF‐1: Ages 16–24 (182–780 ng/ml), Ages 25–39 (114–492 ng/ml), Ages 55 and older (71–290 ng/ml), Thyroxin (T4) 5–12 µg/ml, TSH (µU/ml) 0.5–4.0 μU/ml, LH: females, follicular (3.9–12.0 mIU/ml), mid‐cycle (2.9–9.0 mIU/ml), luteal (1.5–7.0 mIU/ml); FSH: females, follicular (1.5–8.0 mIU/ml), mid‐cycle (2.0–8.0 mIU/ml), luteal (0.2–6.0 mIU/ml); PRL: males (42.5–414 ng/dl), females (51.0–580 ng/dl); Testosterone, males (1.95–11.38 ng/ml); Estradiol: females, follicular (57–227 pg/ml), mid‐cycle (127–476 pg/ml), luteal (77–277 pg/ml); hbA1C, non‐diabetic level (<6%), near normal glycemia (6–7%), insufficiently controlled (7–8%), poorly controlled (>8.5%). Serum Total Cholesterol, Desirable ˂200 mg/dl Borderline‐high 200–239 mg/dl High >239 mg/dl.

### 3.2 Variant identification and sequence analysis

**FIGURE 3:**
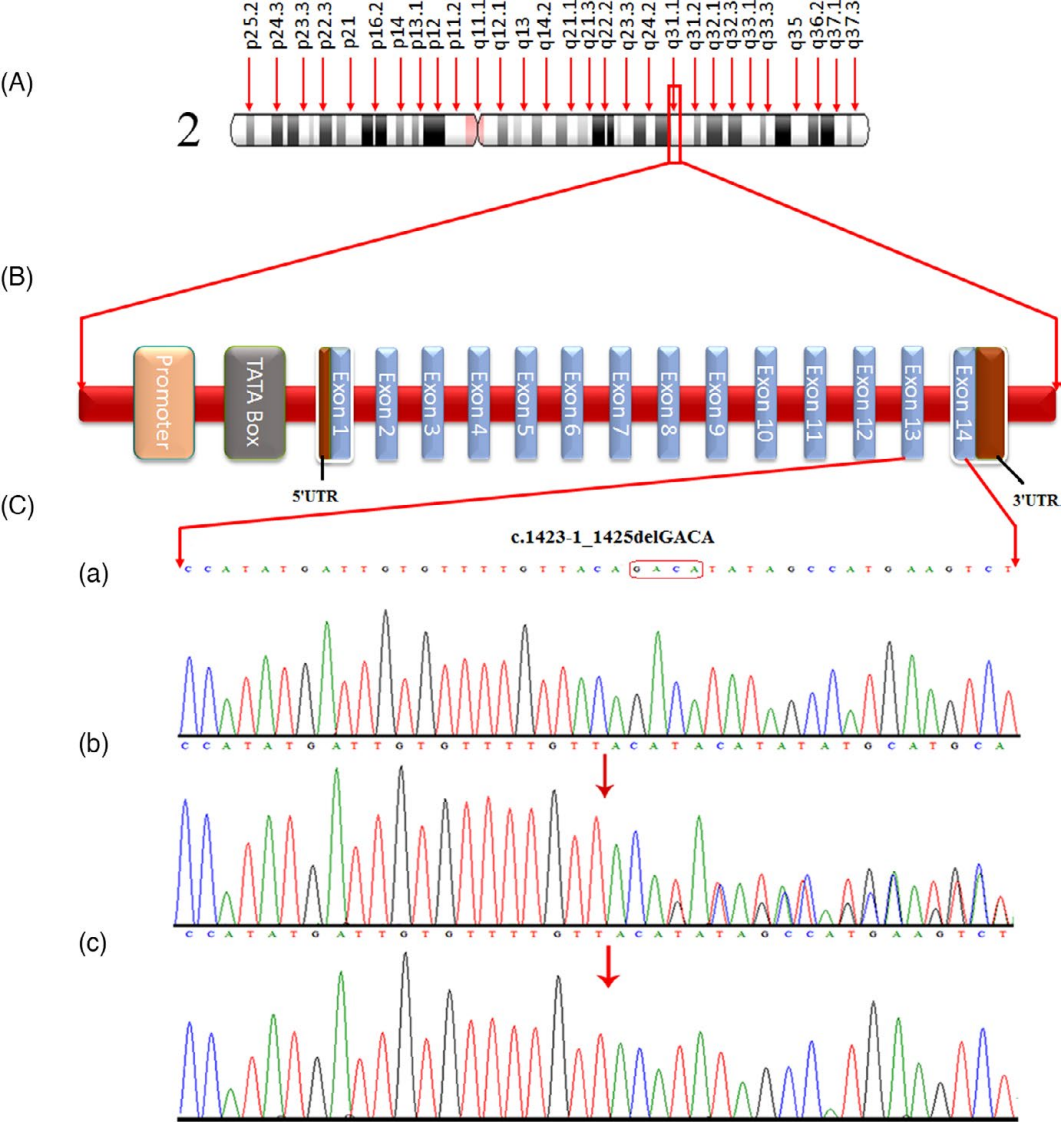
Sanger sequence chromatogram of DCAF17; the upper panel shows the homozygous wild allele in the unaffected brothers (IV‐6), the middle panel shows the heterozygous mutant allele in the carriers (III‐1 and III‐2) and the lower panel shows the homozygous wild‐type allele in the unaffected sisters (IV‐1, IV‐2, IV‐3, IV‐4, and IV‐5)

Based on autosomal recessive mode of inheritance and consanguinity, rare homozygous pathogenic variants, including missense variants, splice acceptor and donor site variants, nonsense and frameshift variants in genes that are known for related phenotypes, as per CENTOGENE data were considered. This results in identification of a variant NM_025000.3:c.1423‐1_1425del. This variant, chr2:172337483_172337486delGACA, is located at splicing site (c.1423‐1_1425delGACA) in *DCAF17* (NM_025000.3) gene mapped on chromosomes 2q22.3–2q35. The sequence variant c.1423‐1_1425del has never been reported in public databases, including gnomAD, 1000 genome, Exome sequencing project, CentoMD, and 100 inhouse Pakistani exomes. Also, the variant flanking sequence is highly conserved in different species, which is confirmed by PhastCons^1^ and PhyloP (0.3 to 5.4) scores. Based on the rarity of the variant, conservation of the sequence, and previous reports of *DCAF17* in Woodhouse‐Sakati syndrome,^2^, ^4^, ^5^ the variant was considered the potential candidate for the disease phenotypes in the studied family. Sanger sequencing analysis confirmed the co‐segregation of the variant with the disease phenotypes in the family by using the primer sequences, left primer 5’‐ AGCAGCGTTGGCAATAGAAT −3’ and right primer 5’‐ GAGAATGTGCCATGCAGATAA‐3’ in exon no 14 of *DCAF17* gene (Figure 3).

## 4 DISCUSSION

In affected members of the family under study, neuroendocrine and ectodermal features of variable degrees were observed, including fragile sparse and fine scalp hair, scanty eyebrows, and absence of axillary and pubic region hair. However, eyelashes in affected homozygotes were indistinguishable from normal individuals in the family. Mild‐to‐moderate sensorineural hearing loss and intellectual disability were also detected. Extrapyramidal features of variable degree were detected in affected individuals, especially in the affected brother (IV‐5), which includes dystonia, choreoathetosis, dysarthria, dysphagia, spastic quadriplegia, myogenic contractures, seizure, and microcephaly. The feature of delayed speech and language development was common in all the mutant homozygotes. The affected individuals, IV‐3 and IV‐5, also display blepharospasm. Among the endocrine features, all the affected members presented hypogonadism, failure of secondary sexual characters development, and widened intermammillary distance. Breast hypoplasia, infantile uterus with absent menstruation, and diabetes mellitus were noted in elder affected sisters (IV‐1 and I V‐2). Affected individuals also exhibited variable degrees of facial dysmorphism, which is rarely reported in individuals with WSS phenotypes. Flattened T wave was recorded in ECG of elder affected sisters (IV‐1 and IV‐2). For laboratory tests, samples were available of three affected members, including two females (IV‐1 and IV‐2) and one male (IV‐3). The laboratory tests showed perturbance in LH, FSH, PRL, estradiol, IGF‐1, and serum cholesterol in affected sisters IV‐1 and IV‐2, respectively. However, the level of thyroxin and TSH was normal. In affected brother IV‐3, most laboratory tests showed normal range, such as LH, FSH, thyroxin, TSH, Testosterone except elevated serum cholesterol, and decreased IGF‐1 levels (Table 1).

The mutation detected in this study is a deletion mutation at the splicing acceptor site, most probably leading to exon skipping. However, if the splicing does occur then, due to the deletion of the two nucleotides from the coding region, there will be a frameshift. In each case, probably a truncated protein will be translated, causing lack/altered residues after 474 amino acids, resulting in a functionless protein. It is also predicted to be a disease‐causing by mutation taster. Previously, 24 different variants are reported in the *DCAF17* gene in people of different ethnicities, including Saudis, Turkish, Pakistani, Indian, Italian, and French. Interestingly, this is the first splicing‐site deletion mutation ever reported.

The exact mechanism of action of the disease is still unidentified because of a deficiency of knowledge regarding the precise functioning of the DCAF17 protein. However, based on the hypothesis of Alazami,^1^ the *DCAF17* mutations disrupt the cell nucleolus, which, in turn, causes inadequacies in the synthesis of ribosomes, cell cycle regulation, process of cellular aging, signal recognition, processing of small‐RNA, transport of mRNA, and even in apoptosis.^9^ Distraction of these processes in our patients may result in the development of WSS phenotypes. Its expression is abnormal in different body parts, including skin, liver, brain, and seminiferous tubules, without any conclusive in vitro demonstration, in islet cells. Studies infer possible defects of islet cells caused by low level of C‐peptide and pancreas atrophy, but further animal‐based studies are obligatory to explain these findings.^16^ Similarly, the *DCAF17* knockout mice generated by gene targeting show that the *DCAF17* mice produced a decreased number of sperm, with abnormal shape and significantly low motility. Histological examination of the testis shows impairment of spermatogenesis with the presence of vacuoles and sloughed cells in the seminiferous tubules. Disruption of *DCAF17* caused asymmetric acrosome capping, impaired nuclear compaction, and abnormal round spermatid to elongated spermatid transition. These studies demonstrate the crucial role of *DCAF17* in spermiogenesis and infertility.^17^ Despite this, the pathogenic mechanism behind the clinical heterogeneity associated with various mutations has not been well investigated. More research is needed to determine the exact pathogenic processes by which these genes affect distinct tissues.

Mutation identification in the *DCAF17* gene is useful tool, for confirming the diagnosis of WSS, predominantly in those young patients, in whom the distinctive symptoms of this syndrome have not yet been fully established. Prevalence of a high rate of consanguineous marriages among Pakistani community increases the relative risk of such rare autosomal recessive disorders in the coming generations. Despite the absence of precise treatment, genetic testing helps to confirm the diagnosis, allow a more accurate prognosis and proper genetic counseling of the patients and their family members.

## CONFLICTS OF INTEREST

The authors declare that they have no conflict of interest.

## AUTHOR CONTRIBUTIONS

Conceptualization, Saadullah Khan and Rubina Nazli; methodology, Fozia Fozia and Sher Alam Khan; software, Fozia Fozia, Sher Alam Khan, and Ijaz Ahmad; Validation, Sher Alam Khan, Rubina Nazli, and Fozia Fozia; formal analysis, Fozia Fozia; investigation, Fozia Fozia; resources, Ijaz Ahmad and Noor Mohammad; data curation, Rubina Nazli; writing—original draft preparation, Fozia Fozia and Khadim Shah; writing—review and editing, Fozia Fozia, Khadim Shah, Saadullah Khan, and Rubina Nazli; Visualization, Saadullah Khan; supervision, Saadullah Khan and Rubina Nazli; project administration, Saadullah Khan; funding acquisition, Amal Alotaibi; writing, review and editing

All authors have read and agreed to the published version of the manuscript.

## Data Availability

All the data used in this manuscript can be obtained upon request.
